# Mutations Associated with Rifampicin Resistance in *Mycobacterium tuberculosis* Isolates from Moroccan Patients: Systematic Review

**DOI:** 10.1155/2020/5185896

**Published:** 2020-10-09

**Authors:** Rkia Eddabra, Mounsef Neffa

**Affiliations:** ^1^Higher Institute of Nurses Professions and Health Techniques of Guelmim, Guelmim, Morocco; ^2^Laboratory of Microbial Biotechnology and Plant Protection, Faculty of Sciences, University Ibn Zohr, Agadir, Morocco; ^3^Laboratory of Bioresources, Biotechnology, Ethnopharmacology, and Health Team: Applied Biochemistry to the Valorization of Bioresources, Mohamed 1st University. Faculty of Sciences, Oujda, Morocco

## Abstract

**Background:**

In recent years, the treatment of tuberculosis has been threatened by the increasing number of patients with drug resistance, especially rifampicin resistance, which is the most effective first-line antibiotic against *Mycobacterium tuberculosis*.

**Methods:**

We performed a systematic review of the literature by searching the PubMed database for studies of rifampicin-resistant *Mycobacterium tuberculosis* (MTB) isolates from Moroccan patients, published between 2010 and 2020. The aim of this review was to quantify the frequency of the most common mutations associated with rifampicin resistance, to describe the frequency at which these mutations co-occur. Identified studies were critically appraised according to the Quality Assessment of Diagnostic Accuracy Studies-2 (QUADAS-2) tool.

**Results:**

6 studies met our inclusion criteria. Results show that 99.36% of MTB isolates had a single-point mutation, and the most commonly mutated codon of *rpo*B gene is 531 with 70.33% of phenotypically resistant strains. However, 10.38% of MTB strains phenotypically resistant to RIF did not exhibit any mutation in the *rpoB* gene.

**Conclusion:**

Identification of a resistance-associated mutation to rifampicin can be a good marker of drug-resistant TB, but lack of a mutation in the target sequence must be interpreted with caution.

## 1. Background

Drug-resistant tuberculosis (TB) is a major public health problem that threatens progress made in fighting TB [1]. Rifampicin (RIF) is one of the most potent antituberculosis drugs, due to its fast-acting bactericidal effects [2]. Resistance to rifampicin involves alterations on the gene encoding the *β*-subunit of the RNA polymerase (*rpoB* gene) [3]. Several studies have shown that more than 95% of the RIF-resistant strains are present within an 81 base pair region of the *rpoB* gene called the RIF-resistance determining region (RRDR) or hot-spot region, which is located between codons 507 and 533 of the *rpoB* gene [4, 5]. The types of mutations include single-nucleotide changes that result in single amino acid substitutions (93%), in-frame deletions (4%), and insertions (3%) [6–8].

The World Health Organization (WHO) estimated that in 2018, there were about half a million new cases of rifampicin-resistant TB (RIF^R^-TB), of which 78% had multidrug-resistant TB (MDR-TB) (defined as resistance to at least isoniazid and rifampicin, with or without resistance to other first-line drugs (FLD)) and only 25% were treated [9]. The increasing emergence of drug-resistant strains of TB is due to treatment defaulters and other challenges (delays in initiating treatment, inadequate treatment, new infections…) [10, 11]. The WHO reported that MDR/RIF^R^ tuberculosis was globally found among 18% of previously TB-treated cases and 3.4% of new cases in 2018 [9].

In Morocco, tuberculosis is endemic and a major public health problem and 36 000 cases of TB were notified in 2018, with nearly 150 new cases per 100 000 inhabitants [12]. The incidence rate of MDR/RIF^R^-TB was estimated to be 530 in the year 2018 compared to 160 patients in 2015 [13]. Despite the use of the directly observed treatment short-course strategy for controlling the disease since 1991, the incidence is still increasing, with an alarming increase in the number of MDR-TB with each passing year [14]. In Morocco, it has been reported that MDR-TB was found among 1% of new TB infections and 8.7% of previously treated cases [15].

Rapid detection of rifampicin resistance in clinical isolate will have an obvious patient as well as public health benefits, including early access to the appropriate treatment, and control of *Mycobacterium tuberculosis* (MBT) by reducing the spread of drug-resistant strains [16–18]. Drug susceptibility testing (DST) induces serious delays in the detection of resistance due to the extremely slow growth of MTB (takes several weeks to months) [19]. To overcome the limitations of phenotypic methods, various molecular detection methods have been recommended and endorsed by the WHO [20]. These methods can speed up MBT identification and drug susceptibility testing, and thus lead to faster and more specific treatment of patients [20].

To date, no systematic review has attempted to assess the prevalence of the most common mutations associated with RIF resistance in Morocco. It is important to understand the frequency and geographic distribution of mutations associated with RIF resistance. The purpose of the present systematic review was to quantify the most common mutations associated with *rpoB* gene in *Mycobacterium tuberculosis* isolates from Moroccan patients.

## 2. Methods

### 2.1. Literature Search

A search in PubMed was conducted on all peer-reviewed publications evaluating mutations in rifampicin-resistant isolates of *M. tuberculosis.* The search was limited to studies published between 2010 and 2020. The search was performed through PubMed using the following key words individually and as an exhaustive combination applying the AND operator: “rifampicin,” “resistance,” “tuberculosis,” “mutations,” and “Morocco” in various combinations.

**Figure 1:**
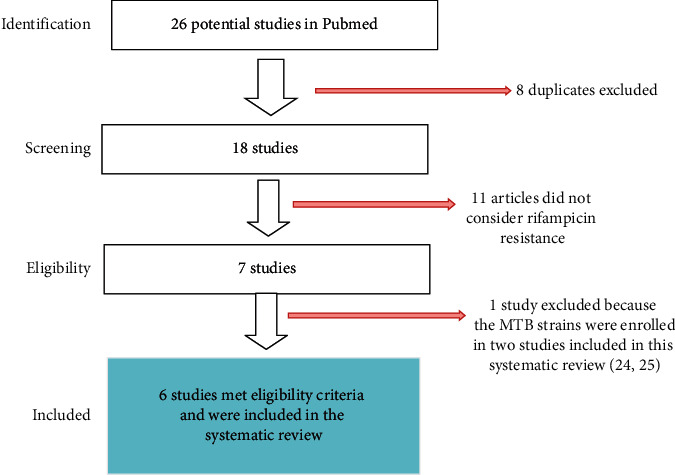
Flow diagram illustrating study selection.

We limited the review to clinical studies written in English. The selection of articles for review was done in three stages: looking at the titles alone, then abstracts, and then full-text articles Figure 1.

### 2.2. Publication Selection Criteria

All studies that met the following inclusion criteria were selected:

1. Presented original data
2. Drug resistance confirmed by the drug susceptibility testing (DST) method used as reference standard
3. We included studies that used liquid and/or solid-based media for DST
4. Assessed mutations in *rpoB* gene in clinical MTB strains (RIF^R^ and/or MDR)

Duplicate publications of the same study were excluded from the analysis.

### 2.3. Data Acquisition

Data extracted from each publication that met the inclusion criteria were as follows: primary author, publication year, geographic origin of specimens, year (s) of specimen collection (the study period), sex ratio, age range, sample size, phenotypic drug susceptibility testing method, genotypic testing method, total number of resistant, and susceptible isolates tested.

Individual isolate mutation information included the following: location of gene mutation, amino acid and nucleotide changes, and frequency of mutations in the *rpoB* gene. Data were recorded and compiled using Excel software (Microsoft, Redmond, WA).

### 2.4. Calculation of Cumulative Mutation Frequencies

Cumulative mutation frequency in resistant isolates was calculated as the number of resistant isolates in which the mutation was found, divided by the total number of phenotypically resistant isolates tested across studies [21].

### 2.5. Assessment of Individual Studies' Methodological Quality

We assessed the quality of studies using the Quality Assessment of Diagnostic Accuracy Studies-2 (QUADAS-2), a validated tool for diagnostic studies [22]. The QUADAS tool consists of 4 key domains including patient selection, choosing index test, reference standard, and optimizing flow and timing [22].

Each domain is assessed in terms of risk of bias, and the first three domains are also assessed in terms of concerns regarding applicability [23]. If the answers to all signaling questions for a domain were “yes”, the risk of bias is judged as “low”; if any signaling question in a domain was “no,” risk of bias is judged as “high.” The unclear bias is used if insufficient information was supplied [22]. Applicability was judged as low, high, or unclear with the similar criteria.

## 3. Results

### 3.1. Study Selection

Initial search parameters identified 18 studies published between 2010 and 2020. After full-text screen, 11 were excluded and they did not meet inclusion criteria (Figure 1), and 7 publications met all eligibility criteria. However, one study was excluded because the MTB strains were enrolled in two other studies included in this systematic review [24, 25], and finally, 6 studies were included in the review.

### 3.2. Methodological Quality of Studies

The quality assessment for each separate study is given in the supplementary material (available here). None of the studies had high risk of bias in three QUADAS-2 domains (patient selection, index test, and reference standard). All studies were of unclear risk for patient selection bias. One study was at high risk for flow and timing bias, resulting from the fact that not all selected patients were included in the genotypic drug susceptibility analysis. Most of the studies were at either low or unclear risk for the index test and reference standard bias. Regarding applicability, all studies were at low risk for patient selection and were at unclear risk of the index test, and one study had a high risk for the reference standard, resulting from the fact that it interpreted the reference test while knowing the results of the genotypic drug susceptibility.

### 3.3. General Characteristics of Included Studies

The characteristics of included studies are given in Table 1. Of the 6 studies included, the earliest was published in 2013, 4 (66.66%) were published in 2017 (2 studies) and in 2018 (2 studies). For all studies, the reported geographic origins of these strains were from different Moroccan cities.

A total of 1589 clinical isolates were collected from TB confirmed pulmonary patients [24, 26, 28, 29], and 203 isolates were collected from suspected TB patients [25, 27]. 25.55% (458/1792) of TB patients were new cases, 30.25% (542/1792) were previously treated, and 1.84% (33/1792) of patients were under treatment. The information regarding 42.35% (759/1792) of patients was not available.

### 3.4. Mutations in the *rpoB* Gene

Conventional drug susceptibility testing (DST) was performed on a Lowenstein–Jensen (L-J) medium [24–26, 29] or using the proportion method [27, 28]. Results showed various phenotypic resistance profiles (Table 2); out of total 1792 MTB specimens, 874 (48.77%) were susceptible for all first-line drugs, 248 (13.84%) were isoniazid resistant (INH^R^), 436 (24.33%) were RIF resistant, and 234 (13.05%) were MDR. Among 918 MTB strains phenotypically resistant to rifampicin and/or isoniazid (248 INH^R^, 436 RIF^R^, and 234 MDR), 495 (238 RIF^R^, 234 MDR, and 23 INH^R^) were subjected to *rpoB* mutation analysis by various genotyping resistance tests: PCR and DNA sequencing [25, 26], qPCR-HRM [29], and rifoligotyping [24], where the genotypic tests were done after culture and the DNA was immediately used or stored at −20°C until use; however, for GenoType® MTBDR*plus* V2.0 [27, 28], the assay was applied directly in the sputum specimens or after solid culture (Table 2). In comparison to phenotypic data, 49 (10.38%) (8 MDR and 41 RIF^R^) MTB strains phenotypically resistant to RIF did not exhibit any mutation in the *rpoB* gene (Table 2).

Mutation data (mutation location, original amino acid and nucleotide, and mutated amino acid and nucleotide) and cumulative frequencies in the *rpoB* gene are reported in Table 3. Twenty-one different types of mutations were identified (dual mutation at codons 516 and 531 was counted as one type).

We observed that there was a diverse profile of the *rpoB* gene mutation (19 different missense mutations and 2 deletions at codons 518 and 520). 99.36% (*n* = 496) isolates had a single-point mutation, and the most commonly occurring mutation in RIF-resistant isolates, at position 531 of the *rpoB* gene, was identified in accounting for 332/472 (70.33%) phenotypically resistant strains. 3 isolates had mutations in both codons 516 and 531.

Other notable mutations identified in *rpoB* included the position 516 and 526, which were found in 8.26% and 8.05% of resistant strains, respectively. The remaining mutations have been found in a limited number and had frequencies of less than 1% among phenotypically resistant isolates.

## 4. Discussion

Drug resistance in *Mycobacterium tuberculosis* is associated with chromosomal mutations in chromosomal genes (e.g., *katG*, *inhA*, *rpoB*, *pncA*, *embB*, *rrs*, *gyrA*, and *gyrB*), rather than by plasmids or transposons [30]. First-line drugs, commonly used for treating tuberculosis such as isoniazid (INH), rifampicin (RIF), pyrazinamide (PZA), and ethambutol (EMB), are becoming ineffective due to mutations in certain genes [31], and their removal through resistance from the anti-TB drug armamentarium has serious implications.

Tuberculosis (TB) strains that are resistant to the first-line TB treatment regimens are more difficult to treat than drug-susceptible ones [32], entails extended chemotherapy (up to 2 years of treatment), with medicines that are expensive and toxic, and higher rates of treatment failure and death [13, 33]. Rapid diagnosis and accurate detection of all forms of drug-resistant tuberculosis is a key factor for effective patient care and for reducing and containing the spread of these resistant strains [34].

Rifampicin is one of the most effective anti-TB antibiotics used to combat infections by *M. tuberculosis.* Resistance of *M. tuberculosis* to rifampicin is mainly due to mutations in the hot-spot region of the *rpoB* gene [4, 5], it has been shown that the emergence of rifampicin resistance occurs rarely when compared to other antibiotics [35], and around 90% rifampicin-resistant cases are also resistant to isoniazid [36]. Therefore, rifampicin resistance is considered as a surrogate marker for drug resistant and for MDR-TB.

In the present review, 495 *M. tuberculosis* isolates from individuals with TB in different Moroccan cities were tested for their drug sensitivity against the first-line anti-TB drugs using different molecular assays, of which 472 (RIF^R^ and/or MDR) were screened for mutations associated with resistance to rifampicin. The results show that mutations in codons 516 (8.26%), 526 (8.05%), and 531 (70.33%) are the most associated mutations with rifampicin resistance. These mutations have already been reported and are in concordance with previous published studies for strains from other parts of the world, which reflect a global pattern [37–39].

Notwithstanding the fact that genotypic drug susceptibility testing has a high sensitivity and specificity but is still unable to detect all the resistance, especially in strains with novel or unknown resistance mechanisms [40, 41]. In this review, 49 isolates with confirmed phenotypic RIF-resistance do not harbor any known mutation in the *rpoB* gene, which may be explained by the fact that RIF resistance-conferring mutations are present elsewhere in the *rpoB* gene (such as a V146F and I572F) [42, 43], suggesting that the nature and frequency of mutations in the *rpoB* gene vary considerably, between different geographical regions [44], by the fact that not all the mutations are targeted by the probes used [45], or as it has been reported that molecular assays still have some drawbacks, such as product cross contamination which is a major problem leading to false positive results [20]. The reason for this cross contamination has not been elucidated properly [46], but it may be due to laboratory procedures (protocol for pretreatment, DNA extraction, and detection of the amplification product) [47]. Therefore, failure to detect RIF-resistance with rapid molecular tests (about 10% of cases in this study), added to the risk factors for unsuccessful TB treatment and MDR-TB development, could risk gains made in the fight against TB.

This is the first systematic review to describe the frequency, location, and type of *rpoB* mutations in RIF-resistant isolates of *M. tuberculosis* in Morocco. However, limitations of the current review included a relatively small number of studies satisfying the selection criteria, most of the selected studies had small sample sizes, and information on prior treatment status was unfortunately often lacking; we can therefore not provide reliable prevalence rates for primary or persisting infections. Several studies have shown that mutations in the *rpoB* gene were different from one region of the world to another TB endemic region [44, 48]. Therefore, further studies on the characterization of drug-resistant strains are needed to describe with enough depth and clarify the behavior of the mutations found in the *ropB* gene analyzed in the isolates circulating in Morocco.

## 5. Conclusion

The most commonly mutated codons in RIF-resistance determining region (RRDR) of the *rpoB* gene are 531, 526, and 516, although there were other missing mutations. Therefore, research on mutations, especially screening the *rpoB* gene associated with rifampicin resistance, should be extended in a larger population and in different Moroccan cities, which can reveal new region-specific mutations that may occur outside the target region.

## Figures and Tables

**Table 1:** Characteristics of studies included in the review.

	Studies included in the review	Study area	Study population	No. of clinical MTB isolates	Year of collection	Sex ratio	Age range	Sample size
1	Oudghiri et al. 2018 [26]	Different cities in Morocco	Confirmed pulmonary TB	703	2010–2102	3.2	3–62 (80% were in the age group of 16–45 years old)	New: 228
								Previously treated (drug relapse and failure): 264
								Under treatment: 33
								Not available: 178

2	Karimi et al. 2018 [27]	Different cities located in the northern region	Suspected TB patients	70	2013-2014	5.36	16–72	New: 42
								Previously treated (drug relapse and failure): 14
								Unknown treatment history: 14

3	Ennassiri et al. 2017 [28]	Different cities in Morocco	Pulmonary TB	319	2013–2015	4.4	Median age of 35.6.	New: 88
								Previously treated: 231

4	Bentaleb et al. 2017 [29]	Different cities in Morocco	Confirmed pulmonary TB	67	NA	NA	NA	NA

5	Chaoui et al. 2014 [24]	Different cities in Morocco	Pulmonary TB	500	Isolates of MTB collected over a period of five years	NA	NA	NA

6	Zakham et al. 2013 [25]	Different cities in Morocco	Suspected TB patients	133	NA	2.64	15–80 (median age 38)	New: 100
								Previously treated (drug relapse or failure, chronic cases): 33

NA: not available, TB: tuberculosis, and MTB: *Mycobacterium tuberculosis*.

**Table 2:** Phenotypic and genotypic drug susceptibility results.

		Conventional drug susceptibility testing (DST)				Genotypic drug susceptibility method			Comparison of phenotypic and genotypic resistance (INH^R^/RIF^R^)
Studies included in the review	Isolates (*n*)	Susceptible for all first-line drugs	INH^R^	RIF^R^	MDR	Molecular assay	Sample processing and DNA extraction	Isolates (*n*)	
Oudghiri et al. 2018 [26]	703	221	194	198	90	PCR and DNA sequencing (performed only for MDR: 90)	DNA was prepared from scraped colonies suspended in distilled water, followed by heat inactivation	84	6 MDR isolates contained no mutations in the sequenced region (157 bp)
							DNA was immediately used or stored at −20°C until use		

Karimi et al. 2018 [27]	70	19	13	12	26	GenoType® MTBDR*plus* V2.0 (performed for all resistant strains: 51)	The assay was applied on direct sputum specimens and on culture isolates	47	4 (1 INH^R^ and 3 RIF^R^) phenotypically resistant strains did not exhibit any mutation using GenoType® MTBDR*plus* assay

Ennassiri et al. 2017 [28]	319	172	31	9	107	GenoType® MTBDR*plus* V2.0 (performed for RIF^R^ and MDR: 116)	The assay was performed on isolates after solid culture or directly on decontaminated sputum specimens	98	18 RIF^R^ samples were missing wild-type probes with no gain in mutation probes

Bentaleb et al. 2017 [29]	67	22	—	45	—	qPCR-HRM (120 pb) (performed for RIF^R^ strains: 45)	DNA was extracted and purified using QIAamp DNA mini kit according to the manufacturer's protocol	40	5 RIF^R^ strains contained no mutation in the *rpoB* amplified region and were classified as phenotypically RIF-resistant isolates
							DNA was stored at −20°C until use		

Chaoui et al. 2014 [24]	500	346	—	154	—	RIFO (performed for RIF^R^ strains: 154)	DNA was prepared from scraped colonies suspended in 1x TE buffer, followed by heat inactivation	140	14 RIF^R^ isolates that were phenotypically resistant did not exhibit any point mutation in the hot-spot region of the *rpoB* gene
							DNA was stored at −20°C until use		

Zakham et al. 2013 [25]	133	94	10	18	11	PCR and DNA sequencing (performed for all resistant strains: 39)	Specimens decontaminated by N-acetyl-l-cysteine were first thawed and centrifuged. For each specimen, the pellet was treated by heat shock	33	6 (3 INH^R^, 1 RIF^R^, and 2 MDR) strains did not exhibit any point mutation in the amplified regions (*rpoB* and *katG* genes and *inhA* promoter region)
							DNA was immediately used or stored at −20°C until use		

Total	1792	874	248	436	234	—	—	442	53 (4 INH^R^, 8 MDR, and 41 RIF^R^)

*n*: number; INH^R^: isoniazid resistant; RIF^R^: rifampicin resistant; RIFO: rifoligotyping; qPCR-HRM: quantitative polymerase chain reaction-high-resolution melting; MDR: multidrug resistant.

**Table 3:** Global frequency of mutations identified in the *rpoB* gene promoter of RIF^R^ of MTB isolates.

Position	511	513			516			518	520	522	526							527	531		Dual mutation	Unknown mutation
Type of mutation	CTG > CCG	CAA > CCA	GAA > CTA	CAA > CTA	GAC > GTC	GAC > TAC	GAC > CAC	D^*∗*^ AAC	D^*∗*^ CCG	TCG > TTG	CAC > TAC	CAC > CTC	CAC > TGC	CAC > CGC	CAC > AGC	CAC > AAC	CAC > GAC	AAG > CAG	TCG > TTG	TCG > TGG		
Amino acid change	Leu/Pro	Glu/Pro	Glu/Leu	Glu/Leu	Asp/Val	Asp/Tyr	Asp/His	—	—	Ser/Leu	His/Tyr	His/Leu	His/Cys	His/Arg	His/Ser	His/Asn	His/Asp	Lys/Glu	Ser/Leu	Ser/Trp	—	—
Oudghiri et al. 2018 [26], *n* = 90	—	1	—	1	3	2	3	—	—	—	—	—	—	3	4	—	—	—	62	5	—	6
Karimi et al. 2018 [27], *n* = 38	—	—	—	—	4	—	—	—	—	—	—	—	—	—	—	—	8	—	23	—	—	3
Ennassiri et al. 2017 [28], *n* = 116	—	—	—	—	9	—	—	—	—	—	2	—	—	—	—	—	6	—	78	—	3^*∗*^	18
Bentaleb et al. 2017 [29], *n* = 45	—	—	—	—	—	—	—	—	—	—	2	1	3	—	—	—	—	—	32	2	—	5
Chaoui et al. 2014 [24], *n* = 154	1	—	—	—	9	4	—	—	—	2	4	1	1	—	—	1	—	—	109	8	—	14
Zakham et al. 2013 [25], *n* = 29	—	1	1	—	2	2	1	2	1	—	—	—	—	1	1	—	—	1	12	1	—	3
**Total number (*n*** **=** **472)**	**1**	**2**	**1**	**1**	**27**	**8**	**4**	**2**	**1**	**2**	**8**	**2**	**4**	**4**	**5**	**1**	**14**	**1**	**316**	**16**	**3**	**49**
**Cumulative frequencies %**	**0.21**	**0.85**			**8.26**			**0.42**	**0.21**	**0.42**	**8.05**							**0.21**	**70.33**		**0.64**	**10.38**

^*∗*^Dual mutation in position 516 & 531. D^*∗*^Deletion.

## Data Availability

Data presented are properly cited and can be obtained from already published original research articles, which are available on electronic databases (e.g., PubMed).

## Supplementary Materials

Supplementary Materials Table S1: tabular presentation for QUADAS-2 results.

## References

[1] Lytras T., Kalkouni O. (2018). The global tuberculosis epidemic: turning political will into concrete action. *Journal of Thoracic Disease*.

[2] Kim B. J., Kim S. Y., Park B. H. (1997). Mutations in the *rpoB* gene of *Mycobacterium tuberculosis* that interfere with PCR-single-strand conformation polymorphism analysis for rifampin susceptibility testing. *Journal of Clinical Microbiology*.

[3] Ramaswamy S., Musser J. M. (1998). Molecular genetic basis of antimicrobial agent resistance in *Mycobacterium tuberculosis:* 1998 update. *Tubercle and Lung Disease*.

[4] Lai C., Xu J., Tozawa Y., Okamoto-Hosoya Y., Yao X., Ochi K. (2002). Genetic and physio-logical characterization of *rpoB* mutations that activate antibiotic productionin Streptomyces lividans. *Microbiology*.

[5] Zaczek A., Brzostek A., Augustynowicz-Kopec E., Zwolska Z., Dziadek J. (2009). Genetic evaluation of relationship between mutations in *rpoB* and resistance of *Mycobacterium tuberculosis* to rifampin. *BMC Microbiology*.

[6] Telenti A., Imboden P., Marchesi F. (1993). Detection of rifampicin-resistance mutations in *Mycobacterium tuberculosis*. *Lancet*.

[7] Valim A. R., Rossetti M. L., Ribeiro M. O., Zaha A. (2000). Mutations in the *rpoB* gene of multidrug-resistant *Mycobacterium tuberculosis* isolates from Brazil. *Journal of Clinical Microbiology*.

[8] Blanchard J. S. (1996). Molecular mechanisms of drug resistance in *Mycobacterium tuberculosis*. *Annual Review of Biochemistry*.

[9] WHO (2019). *Global Tuberculosis Report*.

[10] Chang K. C., Leung C. C., Tam C. M. (2004). Risk factors for defaulting from anti-tuberculosis treatment under directly observed treatment in Hong Kong. *International Journal of Tuberculosis and Lung Disease*.

[11] Cox H., Dickson-Hall L., Ndjeka N. (2017). Delays and loss to follow-up before treatment of drug-resistant tuberculosis following implementation of Xpert MTB/RIF in South Africa: a retrospective cohort study. *PLoS Medicine*.

[12] WHO (2018). *WHO_HQ_Reports-G2-PROD-EXT-TB Country Profile*.

[13] El Hamdouni M., Bourkadi J. E., Benamor J., Hassar M., Cherrah Y., Ahid S. (2019). Treatment outcomes of drug resistant tuberculosis patients in Morocco: multi-centric prospective study. *BMC Infectious Diseases*.

[14] Tazi L., El Baghdadi J., Lesjean S. (2004). Genetic diversity and population structure of *Mycobacterium tuberculosis* in Casablanca, a Moroccan city with high incidence of tuberculosis. *Journal of Clinical Microbiology*.

[15] Ministère de la santé du Maroc (2018). *Plan stratégique national: Pour la prévention et le contrôle de la tuberculose au Maroc 2018-2021*.

[16] Sethi S., Hao Y., Brown S. M. (2019). Elucidation of drug resistance mutations in *Mycobacterium tuberculosis* isolates from North India by whole-genome sequencing. *Journal of Global Antimicrobial Resistance*.

[17] Fan X. Y., Hu Z. Y., Xu F. H., Yan Z. Q., Guo S. Q., Li Z. M. (2003). Rapid detection of *rpoB* gene mutations in rifampin-resistant *Mycobacterium tuberculosis* isolates in shanghai by using the amplification refractory mutation system. *Journal of Clinical Microbiology*.

[18] Alcaide F., Coll P. (2011). Advances in rapid diagnosis of tuberculosis disease and anti-tuberculous drug resistance. *Enfermedades Infecciosas Microbiologia Clinica*.

[19] Dookie N., Rambaran S., Padayatchi N., Mahomed S., Naidoo K. (2018). Evolution of drug resistance in *Mycobacterium tuberculosis*: a review on the molecular determinants of resistance and implications for personalized care. *Journal of Antimicrobial Chemotherapy*.

[20] Eddabra R., Ait Benhassou H. (2018). Rapid molecular assays for detection of tuberculosis. *Pneumonia (Nathan)*.

[21] Seifert M., Catanzaro D., Catanzaro A., Rodwell T. C. (2015). Genetic mutations associated with isoniazid resistance in *Mycobacterium tuberculosis*: a systematic review. *PLoS One*.

[22] Whiting P. F., Rutjes A. W., Westwood M. E. (2011). QUADAS-2: a revised tool for the quality assessment of diagnostic accuracy studies. *Annals of Internal Medicine*.

[23] Whiting P., Rutjes A. W., Reitsma J. B., Bossuyt P. M., Kleijnen J. (2003). The development of QUADAS: a tool for the quality assessment of studies of diagnostic accuracy included in systematic reviews. *BMC Medical Research Methodology*.

[24] Chaoui I., Atalhi N., Sabouni R. (2014). Rifoligotyping assay: an alternative method for rapid detection of rifampicin resistance in *Mycobacterium tuberculosis* isolates from Morocco. *Biotechnology Biotechnological Equipment*.

[25] Zakham F., Chaoui I., Echchaoui A. H. (2013). Direct sequencing for rapid detection of multidrug resistant *Mycobacterium tuberculosis* strains in Morocco. *Infection and Drug Resistance*.

[26] Oudghiri A., Karimi H., Chetioui F. (2018). Molecular characterization of mutations associated with resistance to second-line tuberculosis drug among multidrug-resistant tuberculosis patients from high prevalence tuberculosis city in Morocco. *BMC Infectious Diseases*.

[27] Karimi H., En-Nanai L., Oudghiri A. (2018). Performance of genotype® MTBDRplus assay in the diagnosis of drug-resistant tuberculosis in Tangier, Morocco. *Journal of Global Antimicrobial Resistance*.

[28] Ennassiri W., Jaouhari S., Sabouni R. (2018). Analysis of isoniazid and rifampicin resistance in *Mycobacterium tuberculosis* isolates in Morocco using genotype® MTBDRplus assay. *Journal of Global Antimicrobial Resistance*.

[29] Bentaleb E. M., El Messaoudi M. D., Abid M. (2017). Plasmid-based high-resolution melting analysis for accurate detection of *rpoB* mutations in *Mycobacterium tuberculosis* isolates from Moroccan patients. *BMC Infectious Diseases*.

[30] Volokhov D. V., Chizhikov V. E., Denkin S., Zhang Y. (2009). Molecular detection of drug-resistant *Mycobacterium tuberculosis* with a scanning-frame oligonucleotide microarray. *Methods in Molecular Biology*.

[31] Prasanna A., Niranjan V. (2019). Classification of *Mycobacterium tuberculosis* DR, MDR,XDR isolates and identification of signature mutation pattern of drug resistance. *Bioinformation*.

[32] Kurz S. G., Furin J. J., Bark C. M. (2016). Drug-resistant tuberculosis: challenges and progress. *Infectious Disease Clinics of North America*.

[33] Chan E. D., Laurel V., Strand M. J. (2004). Treatment and outcome analysis of 205 patients with multidrug-resistant tuberculosis. *American Journal of Respiratory and Critical Care Medicine*.

[34] Günther G. (2014). Multidrug-resistant and extensively drug-resistant tuberculosis: a review of current concepts and future challenges. *Clinical Medicine (London)*.

[35] Htike Min P. K., Pitaksajjakul P., Tipkrua N., Wongwit W., Jintaridh P., Ramasoota P. (2014). Novel mutation detection in *rpoB* of rifampicin-resistant *Mycobacterium tuberculosis* using pyrosequencing. *Southeast Asian Journal of Tropical Medicine Public Health*.

[36] Farooqi J. Q., Khan E., Alam S. M., Ali A., Hasan Z., Hasan R. (2012). Line probe assay for detection of rifampicin and isoniazid resistant tuberculosis in Pakistan. *Journal of Pakistan Medical Association*.

[37] Zaw M. T., Emran N. A., Lin Z. (2018). Mutations inside rifampicin-resistance determining region of *rpoB* gene associated with rifampicin-resistance in *Mycobacterium tuberculosis*. *Journal of Infection and Public Health*.

[38] Somoskovi A., Parsons L. M., Salfinger M. (2001). The molecular basis of resistance to isoniazid, rifampin, and pyrazinamide in Mycobacterium tuberculosis. *Respiratory Research*.

[39] Caws M., Duy P. M., Tho D. Q., Lan N. T., Hoa D. V., Farrar J. (2006). Mutations prevalent among rifampin and isoniazid-resistant *Mycobacterium tuberculosis* isolates from a hospital in Vietnam. *Journal of Clinical Microbiology*.

[40] Yadav R., Saini A., Kaur P., Behera D., Sethi S. (2018). Diagnostic accuracy of genotype® MTBDR*sl* VER 2.0 in detecting second-line drug resistance to *M. tuberculosis*. *International Journal of Tuberculosis and Lung Disease*.

[41] Kivihya-Ndugga L., van Cleeff M., Juma E. (2004). Comparison of PCR with the routine procedure for diagnosis of tuberculosis in a population with high prevalences of tuberculosis and human immunodeficiency virus. *Journal of Clinical Microbiology*.

[42] Siu G. K., Zhang Y., Lau T. C. (2011). Mutations outside the rifampicin resistance-determining region associated with rifampicin resistance in *Mycobacterium tuberculosis*. *Journal of Antimicrobial Chemotherapy*.

[43] Heep M., Brandstätter B., Rieger U. (2001). Frequency of *rpoB* mutations inside and outside the cluster I region in rifampin-resistant clinical *Mycobacterium tuberculosis* isolates. *Journal of Clinical Microbiology*.

[44] Adikaram C. P., Perera J., Wijesundera S. S. (2012). Geographical profile of *rpoB* gene mutations in rifampicin resistant *Mycobacterium tuberculosis* isolates in Sri Lanka. *Microbial Drug Resistance*.

[45] Yadav R. N., Singh B. K., Sharma S. K. (2013). Comparative evaluation of genotype MTBDRplus line probe assay with solid culture method in early diagnosis of multidrug resistant tuberculosis (MDR-TB) at a tertiary care centre in India. *PLoS One*.

[46] Venter R., Minnies S., Derendinger B. (2020). Extract from used Xpert MTB/RIF Ultra cartridges is useful for accurate second-line drug-resistant tuberculosis diagnosis with minimal *rpoB* -amplicon cross-contamination risk. *Scientific Reports*.

[47] Noordhoek G. T., Kolk A. H., Bjune G. (1994). Sensitivity and specificity of PCR for detection of *Mycobacterium tuberculosis*: a blind comparison study among seven laboratories. *Journal of Clinical Microbiology*.

[48] Chen L., Gan X., Li N., Wang J., Li K., Zhang H. (2010). *rpoB* gene mutation profile in rifampicin-resistant *Mycobacterium tuberculosis* clinical isolates from Guizhou, one of the highest incidence rate regions in China. *Journal of Antimicrobial Chemotherapy*.

