# Supplementary material for: Mutations Associated with Rifampicin Resistance in Mycobacterium tuberculosis Isolates from Moroccan Patients: Systematic Review
Source: Interdiscip Perspect Infect Dis. 2020 Oct 9;2020:5185896. doi: 10.1155/2020/5185896 (PMC7568785; doi:10.1155/2020/5185896)
Supplement: Supplementary Materials — Table S1: tabular presentation for QUADAS-2 results. [file 5185896.f1.docx]

**Supplementary material**

**Table S1:** Tabular presentation for QUADAS-2 results

| **Study** | **RISK OF BIAS** | | | | **APPLICABILITY CONCERNS** | | |
| --- | --- | --- | --- | --- | --- | --- | --- |
|  | **Patient selection** | **Index test** | **Reference standard** | **Flow and timing** | **Patient selection** | **Index test** | **Reference standard** |
| Oudghiri *et al.* 2018 [[21](#_ENREF_21)] | ? | ☺ | ☺ | ☹ | ☺ | ? | ☺ |
| Karimi *et al*. 2018 [[22](#_ENREF_22)] | ? | ☺ | ☺ | ☺ | ☺ | ? | ☺ |
| Ennassiri *et al.* 2017 [[20](#_ENREF_20)] | ? | ? | ☺ | ☺ | ☺ | ? | ☹ |
| Bentaleb *et al.* 2017 [[19](#_ENREF_19)] | ? | ☺ | ? | ☺ | ☺ | ? | ☺ |
| Chaoui *et al.* 2014 [[17](#_ENREF_17)] | ? | ? | ? | ☺ | ☺ | ? | ☺ |
| Zakham et al. 2013 [[18](#_ENREF_18)] | ? | ☺ | ☺ | ☺ | ☺ | ? | ☺ |

☺ Low Risk ☹High Risk ? Unclear Risk
